# Effects of endophytic fungi on the secondary metabolites of *Hordeum bogdanii* under alkaline stress

**DOI:** 10.1186/s13568-022-01414-w

**Published:** 2022-06-14

**Authors:** Dan Han, Kai Wang, Feng Long, Wangbin Zhang, Xiang Yao, Shuihong Chen

**Affiliations:** 1 Key Laboratory of Biological Resources Protection and Utilization Corps in Tarim Basin, College of Life Sciences, Tarim University, Xinjiang, 843300 Alar China; 2 College of Animal Sciences, Tarim University, Xinjiang, 843300 Alar China; 3 College of Plant Sciences, Tarim University, Xinjiang, 843300 Alar China; 4 Institute of Botany, Jiangsu Province and Chinese Academy of Sciences, Nanjing, 210014 Jiangsu China

**Keywords:** Endophytic fungi, *Hordeum bogdani*, Alkaline stress, Secondary metabolite, Polyphenol, Flavonoid

## Abstract

It is currently unclear whether the mechanism of endophytic fungi improving the alkali tolerance of *Hordeum bogdanii* affects secondary metabolites. Unveiling this knowledge is crucial for understanding the tolerance mechanism of *H. bogdanii* to alkaline stress. The aim of this study was to investigate how endophytic fungi affect secondary metabolites of *H. bogdanii* under alkaline stress at different concentrations. Endophyte-infected (E +) and endophyte-free (E−) individuals of *H. bogdanii* were used as materials in this study. The method of indoor vermiculite aseptic planting was adopted. After mixed alkali stress treatment, the roots, stems, and leaves of the plants were collected to measure the indicators related to secondary metabolites. The results showed that endophytic fungi improved the alkali resistance of *H. bogdanii* by improving the related indicators of secondary metabolites. endophytic fungi significantly increased the contents of phosphorus, polyphenols, and alkaloids, and the activities of polyphenol oxidase and acid phosphatase, and significantly reduced flavonoid content. The content of polyphenols and alkaloids in stems, polyphenol oxidase activity in stems and leaves, and acid phosphatase activity in leaves were significantly affected. The findings of this study may aid in amplifying the alkali resistance mechanism of endophytic fungi to *H. bogdanii* as well as provide insights into improving the alkali resistance of other plants.

## Introduction

Plant secondary metabolism is the result of plant adaptation to the ecological environment during long-term evolution (Wang et al. 2020). Plant secondary metabolites are diverse, with different properties. According to their chemical structures, they can be divided into phenols, terpenes, and nitrogen-containing organic compounds (Li et al. 2021). Secondary metabolites are natural compounds produced by plants and have a variety of physiological roles (Chen et al. 2009). Stress can significantly promote the synthesis of plant secondary metabolites, which participate in the coordination of the relationship between plants and their environments (Tu 2019). Secondary metabolites have been found to play an important role in drought and salt tolerance in plants (Ahmedl et al. 2015). Endophytic fungi can produce a variety of alkaloids, including organic amines, pyrrolizidines, and ergot alkaloids (Xu et al. 2010). Endophytic fungi in gramineous plants can produce four major alkaloids (i.e., organic amines such as peramine, pyrrolizidines such as loline, indole diterpene derivatives such as lolitrem B, and ergot alkaloids such as ergovaline). These have various biological activities, such as resistance to pathogenic bacteria, resistance to nematodes, and enhancement of plant allelopathy.

*Hordeum bogdanii* is a wild gramineous grass distributed in Xinjiang, Gansu, Qinghai, Inner Mongolia, and other regions of China. It is an important wild germplasm resource and is used as forage grass. It has strong cold tolerance and salt-alkali resistance, and plays an important role in the improvement of desertification and saline-alkali land (Jia 1987; Ma et al. 1998). The endophytic fungi of grasses are a large group that grow and complete all or most of their life cycle within plants, while the grasses do not show external symptoms (Gao and Nan 2007; Bongiornoet al. 2016). The endophytic fungus and host coevolve and have a mutually beneficial relationship (Chen et al. 2018a, b; Tanaka et al. 2021). Endophytic fungi obtain nutrients from the host grass, while simultaneously enhancing the resistance of the grass to biological and abiotic stresses, thereby promoting its ability to adapt to the environment. Examples of this include promoting growth, resisting harm by herbivores and nematodes (Kuldau and Bacon 2008; Behie et al. 2012), enhancing resistance to pathogenic fungi (Li et al. 2008; Xia et al. 2018), increase resistance to drought stress (Clay and Schardl 2022; Swarthout et al. 2009), salt stress (Song et al. 2015a, b; Wang et al. 2019), etc.which is often considered a defensive symbiosis (Clay. 1999; Kauppinen et al. 2016). Endophytic fungi can also increase the acquisition of nutrients, produce plant hormones, increase the photosynthetic capacity of plant tissues, promote the production of antibiotics, and help plants improve their self-defense system. (Noemi and Everlon 2022; Poveda et al. 2021) Some researchers have detected endophytic fungi (*Neotyphodiu*m) in *H. bogdanii* in western China (Li and Sun 1996, 1997; Nan 1996). In addition, a comparative study of *H. bogdanii* plants with endophytic fungi (E +) and without endophytic fungi (E-) in the Altai area of Xinjiang, China, showed that endophytic fungi promoted the growth of host *H. bogdanii* and increased tiller number, biomass, and the forage yield of plants (Nan 1996).

Polyphenols are important, physiologically active, secondary metabolites involved in the regulation of growth, development, and response to adversity in plants. Studies have shown that the level of phenolic compounds in plants is significantly increased under a variety of biological or abiotic stresses (Chen 2020). Polyphenol oxidase activity is related to wheat ear germination resistance (Huang et al. 2021). Flavonoids can be used as regulators in the process of plant growth and development and improve plant resistance to certain adverse stresses (Chu et al. 2007). Moreover, plant endophytic fungi can produce secondary metabolites, such as alkaloids and flavonoids (Su 2018).

Endophytic fungi can promote the production of secondary metabolites (such as alkaloids, flavonoids and phenols) (Noemi and Everlon 2022), However, little information is available on the effects of endophytic fungi on secondary metabolites under alkaline stress. Therefore, it is unclear if the mechanism of endophytic fungi improving the alkali tolerance of the host *H. bogdanii* affects the secondary metabolites. This knowledge is greatly significant for understanding the tolerance mechanism of *H. bogdanii* to alkaline stress. In this study, we examined the content of secondary metabolites and related enzymes involved in the process. Our findings will help to amplify the alkali resistance mechanism of endophytic fungi to *H. bogdanii* and supply a new field for exploring effective ways to improve the alkali resistance of plants.

## Materials and methods

### Plant material

The seeds of *H. bogdanii* were collected from Wensu County, Aksu District, Xinjiang Province, China (80° 76ʹ E, 41° 58ʹ N, H 1514 m). The endophytic fungi of *H. bogdanii* in this region is *Epichloë bromicola* (Zhang et al. 2021). The tillers of a *H. bogdanii* plant with endophytic fungi (E +) were divided into two parts, half of which were soaked with fungicide (carbendazim) for six hours, and then watered with 10 times diluted soaking solution to obtain homogeneous plants without endophytic fungi (E−). The seeds of the E + and E− plants bred in the field were used in this experiment. In the greenhouse of Tarim University (daytime temperature 25 °C, night temperature 15 °C, 14 h light per day), E + and E- *H. bogdanii* grass were planted in plastic flowerpots (pot diameter 13 cm, bottom diameter 8 cm, height 12 cm). Every week, 200 ml of Hoagland nutrient solution was poured in each pot and plants were watered regularly. After the plants were cultured for 6 weeks, the endophytic fungi were detected under a microscope using the sheath aniline blue test method (Li et al. 2008), and the E + and E- plants were determined again.

### Alkali stress treatment

With a Na_2_CO_3_:NaHCO_3_ ratio of 1:1, five concentrations of mixed alkali (25, 50, 100, 150, and 200 mmol/L) were used to treat *H. bogdanii* plants, with no added alkali as the control. Each treatment had five replicates. After 21 days of treatment, fresh plant root, stem, and leaf samples were collected and immediately frozen in a refrigerator at − 80 °C. The remaining samples were dried, crushed, sieved, and stored at room temperature to determine the relevant indicators.

### Related physical and chemical index measurement methods

Phosphorus content: using molybdenum antimony colorimetric method (Song et al. 2015a, b; Liang et al. 2017). Polyphenol content: Folin-Ciocalteu method was used to determine the polyphenol content (Shi et al. 2018). Determination of polyphenol oxidase activity: refer to the colorimetric method of Li Zhongguang et al. (Shi et al. 2018). The contents of flavonoids and alkaloids, and acid phosphatase activity were measured using a kit from Suzhou Keming Biotechnology Co., Ltd.

### Data processing and statistical analysis methods

All data in this paper were calculated by Excel 2016, mapped by Sigmaplot12.5, and Spss19 was used to analyze the significance of the difference. The effects of alkali treatment and endophytic fungi on the contents of phosphorus, polyphenols, flavonoids, and alkaloids, and the activities of polyphenol oxidase and acid phosphatase in *H. bogdanii* were detected by two-way ANOVA, The effects of different alkali treatment concentrations on the contents of phosphorus, polyphenols, flavonoids, and alkaloids, and the activities of polyphenol oxidase and acid phosphatase in *H. bogdanii* were detected by one-way ANOVA. An independent sample t-test was used to detect the differences in the contents of E + and E− phosphorus, polyphenols, flavonoids, and alkaloids, and the activities of polyphenol oxidase and acid phosphatase at the same alkali concentration.

## Results

### Effects of endophytic fungi on phosphorus content in roots of *H.bogdanii* under different concentrations of alkali

**Fig. 1:**
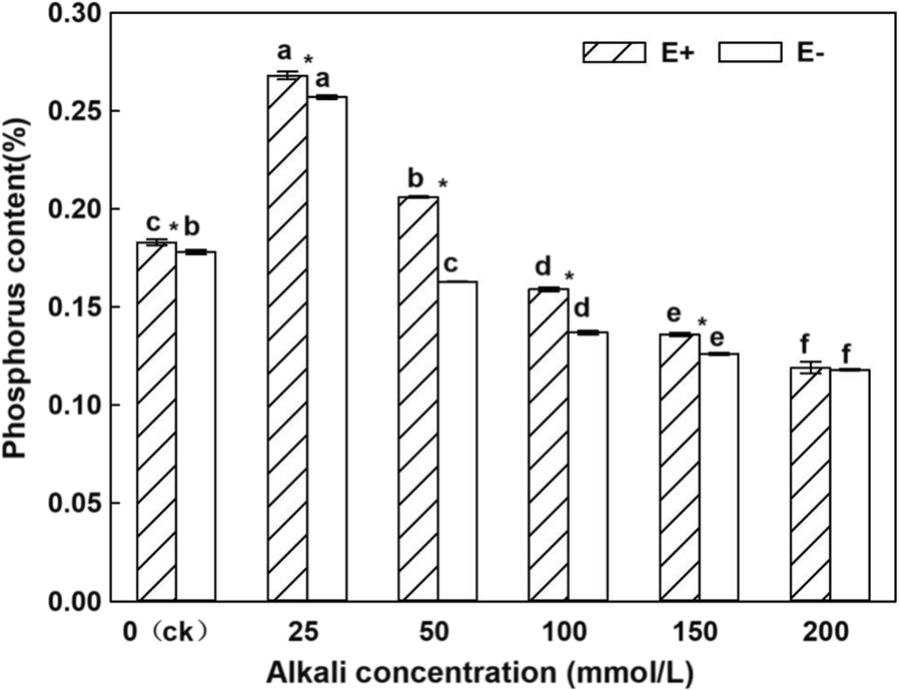
Phosphorus content in E + and E− *Hordeum bogdanii* roots under different concentrations of alkaliIn different treatments, in the same E + or E−, different lowercase letters show significant differences (*P* < 0.05); in the same treatment of E + and E−, significant differences between pairs are indicated by "*" (*P* < 0.05), as shown above.

Under the same treatment, the phosphorus content in the roots of plants with endophytic fungi (E +) was higher than that in the roots of plants without endophytic fungi (E−) (Fig. 1). Although the phosphorus content in the roots of E + and E− plants was not significantly different under the 200 mmol/L alkali treatment, the phosphorus content of E + plants was significantly higher than that of E− plants in the other alkali treatments (*P* < 0.05). The phosphorus content in the roots of *H. bogdanii* plants reached the highest content at an alkali treatment concentration of 25 mmol/L, which was significantly higher than the phosphorus content in the roots of the control plants and showed a significant decreasing trend as the alkali concentration increased (*P* < 0.05). From the two-factor ANOVA table (Table 1), both endophytic fungi and alkali treatment significantly affected the phosphorus content in the roots of *H. bogdanii*, and there was an interaction between the endophytic fungi and alkali treatment.

**Table 1:** Two-factor analysis of endophytic fungi on the phosphorus content, flavonoids, and alkaloids of *Hordeum bogdanii* under alkali stress

		Phosphorus content		Flavonoid content		Alkaloid content	
	df	F	*P*	F	*P*	F	*P*
Endophytic Fungi	1	409.034	0.000	114.922	0.000	1281.122	0.000
Alkali treatment	5	3305.367	0.000	32.157	0.000	549.658	0.000
Endophytic Fungi × Alkali treatment	5	68.466	0.000	6.634	0.001	31.433	0.000

df is the degree of freedom, F is the statistic of F test, *P* is statistical significance, as shown above

### Effects of endophytic fungi on flavonoids and polyphenols of *H. bogdanii* under different concentrations of alkali

**Fig. 2:**
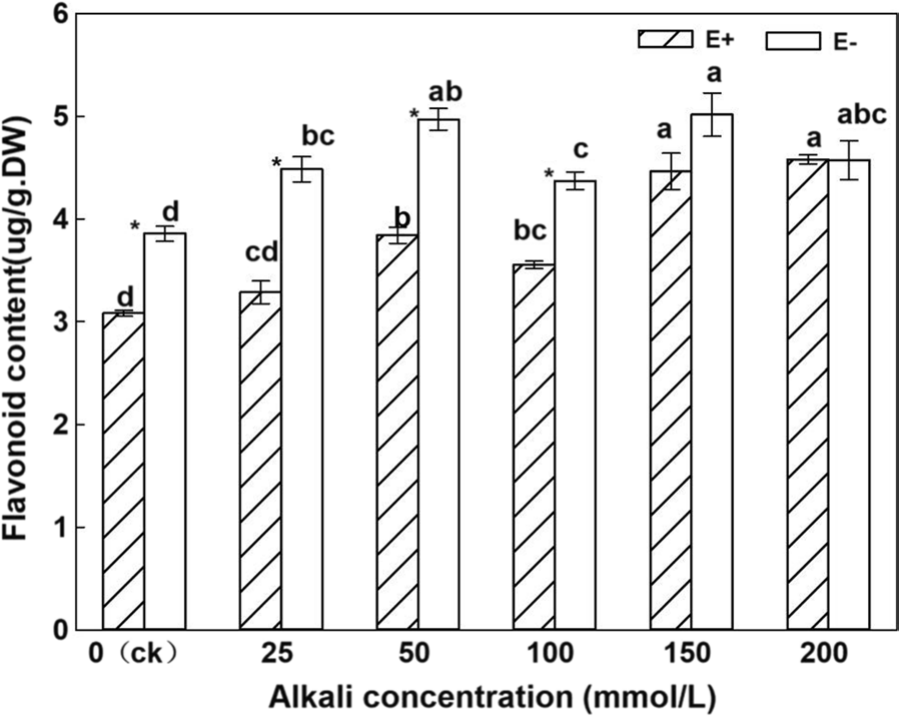
Flavonoid contents in stems of E + and E− *Hordeum bogdanii* under different concentrations of alkali

Under the same treatment, except for the high alkali concentration treatment of 200 mmol/L, the content of flavonoids in the stems of E + plants was significantly lower than that of E− (Fig. 2). With an increase in alkali stress, flavonoid contents increase in E + plants. The flavonoid contents in the E + plants in the 150 mmol/L and 200 mmol/L treatments were the highest, and were significantly higher than that in the control (*P* < 0.05). From the two-factor ANOVA (Table 1), endophytic fungi and alkali treatment significantly affected the flavonoid content in *H. bogdanii *stems, and there was an interaction between endophytic fungi and alkali treatment (*P* < 0.05).

**Fig. 3:**
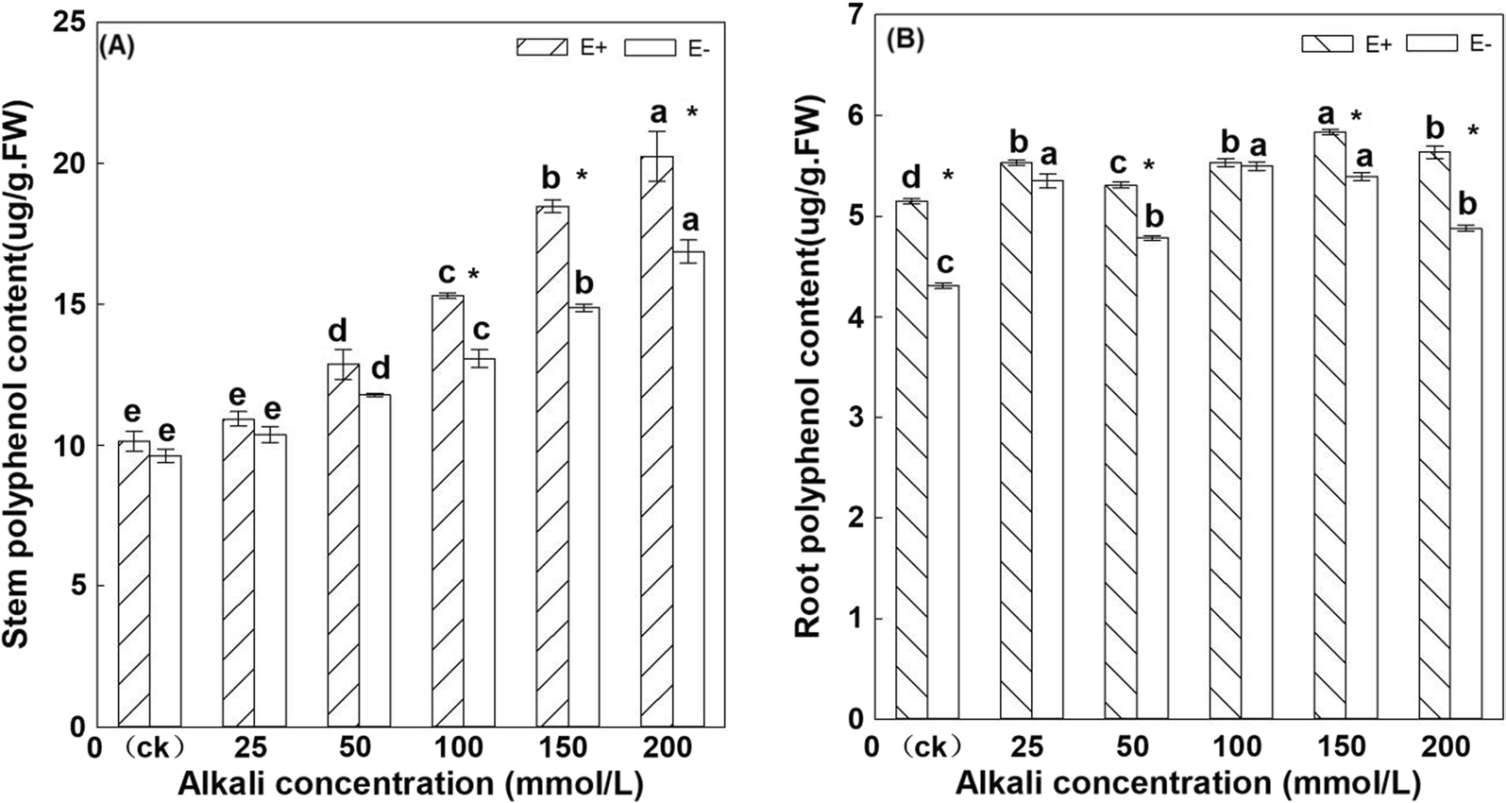
Polyphenol content in the stems and roots of E + and E− *Hordeum bogdanii* under different concentrations of alkali (**A** is the stem, **B** is the root)

Under the same treatment, the polyphenol content in E + *H. bogdanii *stems was higher than that of E−, and the difference was significant when alkali treatments were 100 mmol/L, 150 mmol/L, and 200 mmol/L (*P* < 0.05) (Fig. 3A). The polyphenol content increased with an increase in the alkali treatment concentration, and the polyphenol content in the stem was the highest under the 200 mmol/L alkali treatment. Except for the 25 mmol/L treatment and the control, treatments were not significantly different. The polyphenol content in the stems of the other treatments were significantly higher than that of the control treatment.

Although there was no significant difference in polyphenol content between E + and E− roots in the 25 mmol/L treatment, in the other treatments, polyphenol content in E + plant roots was significantly higher than that in E− plants (*P* < 0.05) (Fig. 3B). The effect of alkali concentration stress in E + roots first lead to an increase and then a decrease in polyphenol content. The polyphenol content was the highest at 150 mmol/L, and the control was the lowest; the polyphenol content in E- root was also the lowest in the control treatment. Therefore, alkali stress also significantly affected the polyphenol content in the roots and stems of *H. bogdanii,* and the results of the two-way ANOVA showed that both the endophytic fungi and alkali treatment significantly affected the content of polyphenols in the roots and stems of *H. bogdanii*, and that there was an interaction between the two (*P* < 0.05).

### Effects of endophytic fungi on alkaloids in the stems of *H.bogdanii* under different concentrations of alkali

**Fig. 4:**
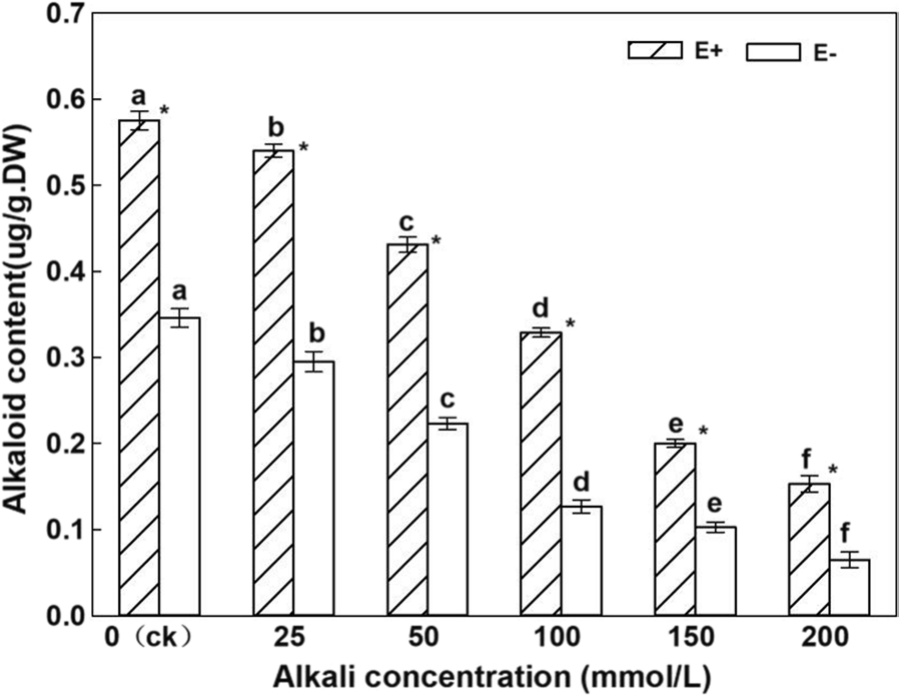
Alkaloid content in the stems of E + and E− *Hordeum bogdanii* under different concentrations of alkali

Under the same treatment, the alkaloid content of E + plants was significantly higher than that of E− plants (Fig. 4). With an increase in the alkali stress treatment concentration, the content of alkaloids in the stems of *H. bogdanii* showed a significant downward trend. With an increase in the alkali treatment concentration, the alkaloid content of E + stems was 22.9, 24.6, 20.8%, 20.1, 9.7, and 8.8% higher than that of the E− stem under the same alkali stress. From the two-factor statistical analysis table (Table 1), both endophytic fungi and alkali treatment significantly affected the alkaloid content in stems, and there was an interaction between endophytic fungi and alkali treatment (*P* < 0.05).

### Effects of endophytic fungi on enzyme activities related to secondary metabolites in stems and leaves of *H. bogdanii* under different concentrations of alkali

#### Effects of endophytic fungi on acid phosphatase in leaves of *H. bogdanii* under different concentrations of alkali

**Fig. 5:**
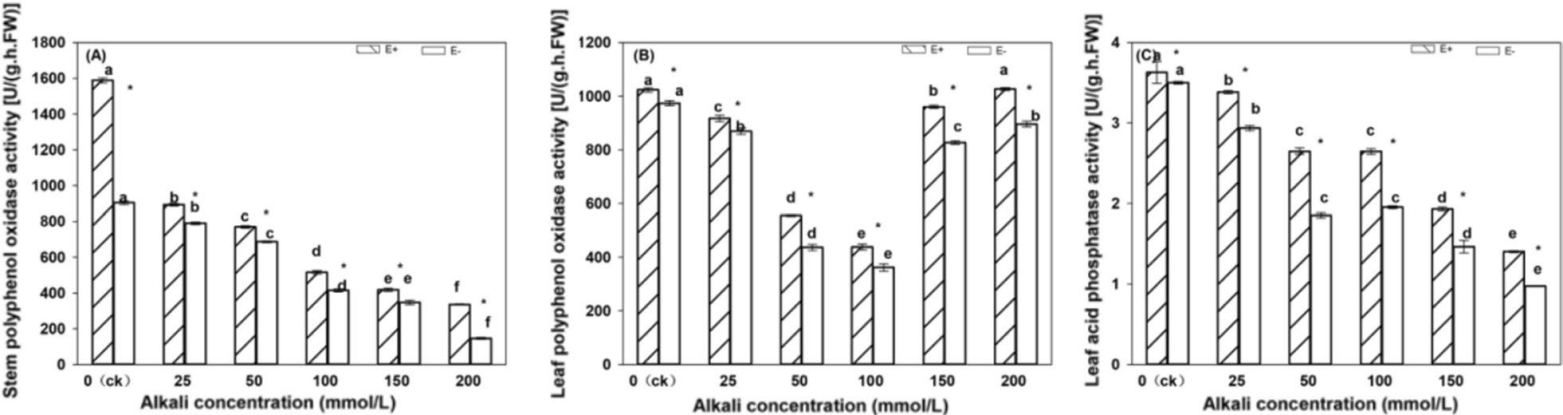
Polyphenol oxidase activity and acid phosphatase activity in stems and leaves of E + and E− *Hordeum bogdanii* under different concentrations of alkali (picture A is polyphenol oxidase activity in stem, picture B is polyphenol oxidase activity in leaves, picture C is acid phosphatase activity in leaves)

Under the same treatment, the acid phosphatase activity in the leaves of E + *H. bogdanii* plants was higher than that of E− (Fig. 5A). Except for the insignificant difference in the control, in all other treatments E + were significantly higher than E− (*P* < 0.05). Acid phosphatase activity showed a decreasing trend with increasing alkali stress concentration, and the alkali treatment was significantly lower than that of the control. From the two-factor analysis table (Table 2), it can be concluded that both endophytic fungi and alkali treatment significantly affected the acid phosphatase activity in *H. bogdanii* leaves (*P* < 0.05), and there was an interaction between the two (*P* < 0.05).

**Table 2:** Two-factor analysis of effect of endophytic fungi on the polyphenol content, and the activities of polyphenol oxidase and acid phosphatase in *Hordeum bogdanii* under alkali stress

		Polyphenol content (stem)		Polyphenol content (root)		Polyphenol oxidase activity (stem)		Polyphenol oxidase activity (leaf)		Acid phosphatase activity	
	df	F	*P*	F	*P*	F	*P*	F	*P*	F	*P*
Endophytic Fungi	1	74.062	0.000	584.955	0.000	1841.759	0.000	284.999	0.000	240.783	0.000
Alkali treatment	5	161.645	0.000	186.247	0.000	3881.38	0.000	1465.891	0.000	576.207	0.000
Endophytic Fungi × Alkali treatment	5	6.593	0.000	49.902	0.000	420.888	0.000	8.817	0.000	12.002	0.000

#### Effects of endophytic fungi on polyphenol oxidase activity in stems and leaves of *H. bogdanii* under different concentrations of alkali

Under the same treatment, the polyphenol oxidase activity in the stems and leaves of the E + plants was significantly higher than that of the E- plants (*P* < 0.05) (Fig. 5B, C). With an increase in the alkali treatment concentration, the activity of polyphenol oxidase in the stems and leaves of *H. bogdani* gradually decreased, and the difference between each alkali treatment was significant. As shown in Table 2, both endophytic fungi and alkali treatment significantly affected the polyphenol oxidase activity in the stems and leaves of *H. bogdani*, and there was an interaction between endophytic fungi and alkali treatment.

## Discussion

Salt and alkalinity affect the physiological and metabolic pathways of plants, similar to other adverse factors. Adversity stress can significantly promote the synthesis of plant secondary metabolites. Secondary metabolism and its products are the material basis of plant response to environmental stress, and its content affects plant resistance to stress (Tu 2019). Polyphenols and flavonoids are important secondary metabolites of plants. To explore whether the mechanism via which endophytic fungi improve alkali tolerance of host *H. bogdanii* influences secondary metabolites, we investigate the contents of secondary metabolites and associated enzymes. In the present study, the infection of endophytic fungi and mixed alkali stress treatment on *H. bogdanii* had significant effects on phosphorus content in roots, flavonoids and alkaloid content in stems, polyphenol content in roots and stems, acid phosphatase activity in leaves, and polyphenol oxidase activity in stems and leaves.

At a concentration of 25 mmol/L, the phosphorus content in the roots of E + and E- plants both reached the highest value and was significantly higher than that of the control. The reason for this may be due to the growth-promoting effect of low alkali concentration, which caused the phosphorus content at this treatment to be significantly higher than that of the control. `Li and Tian (2014) also showed that appropriate salt stress can promote the absorption of phosphorus by plants. Malinowski et al. (2000) found that the accumulation of phosphorus in the roots and stems of Tall fescue (genotype DN2) E + plants was significantly higher than that of E− plants. Acid phosphatase is an important enzyme that regulates phosphorus metabolism in organisms. Acid phosphatase not only participates in the growth and metabolism of organisms and signal transduction pathways, but also enhances plant resistance to phosphorus deficiency, drought, low temperature, water, salt, and other adversities. Wang et al. (2015), Liu et al. (2017), and Javot et al. (2007) also proved that arbuscular mycorrhizal (AM) fungi can significantly promote the absorption of phosphorus by roots after infecting plants, stimulate the secretion and activity of plant phosphatase, and induce an increase in phosphorus content in roots. Xie et al. (2013) also showed that the acid phosphatase activity of plants inoculated with endophytic fungi increased significantly and that the total phosphorus content also increased to a greater extent. The findings of this study also showed that endophytic fungi promoted acid phosphatase activity in *H. bogdanii* leaves, which promoted an increase in phosphorus content. This difference was more obvious under alkaline stress; as the alkali concentration increased both the phosphorus content and acid phosphatase activity showed a downward trend.

Plant polyphenols are an inherent component of many plants. Under normal conditions, their content in plants is very low, but when plants are stimulated by foreign factors, the content of these substances will increase significantly to enhance the resistance to abiotic stress (Zhao. 2004). Studies have shown that the content of polyphenols in plants can increase under adverse conditions (Wang et al. 2007). Phenolic compounds play a vital role in relieving oxidative stress because they are involved in the detoxification of reactive oxygen species (ROS) (Wang et al. 2011). After alkali stress treatment, plants can synthesize phenylalanine, which combines with dopamine to produce alkaloids, and then eliminate ROS (Fan. 2021). It has been reported that in several other grasses (Ryegras、Fescue), endophytic fungal infection increases the content of phenolic compounds in plant roots (Ponce et al. 2009; Vázquez- de-Aldana et al. 2011). The present study also showed that with an increase in alkali stress concentration, the content of polyphenols in the roots and stems of *H. bogdanii* showed an increasing trend, especially in the stems. The content of polyphenols in E + plants was higher than in E− plants. The study also showed that endophytic fungi increase the polyphenol content in the host *H. bogdanii* stem under alkali stress, which help the host to resist alkali stress.

Under alkaline stress, the polyphenol oxidase activity of *H. bogdanii* gradually decreased with the increase in alkali concentration. However, the E + plant was significantly higher than E−, and the control E + was also significantly higher than E−. Previous studies have shown that low concentrations of saline alkali stress can promote polyphenol oxidase activity. Yan et al. (2021) and Zhao et al. (2005) believe that the more alkaline the environment, the faster the enzyme activity decreases. This is consistent with the results of the trend of polyphenol oxidase activity in the stems and leaves of *H.bogdanii* obtained in the present study. Moreover, studies have also shown that when the activity of polyphenol oxidase decreases, the enzymatic reaction is inhibited, leading to an increase in polyphenol content (Zhang et al. 2013). The results of the present study also showed that under high alkali concentrations, the activity of polyphenol oxidase decreased and polyphenol content increased.

Under different concentrations of alkali stress, endophytic fungi had a significant effect on the alkaloid content in the stems of *H. bogdanii*. Alkali treatment reduced the alkaloid content of *H. bogdanii*. It has been reported that the total alkaloid content of plants decreases with an increase in pH (Tang and Chen. 2011), which is consistent with the results of the present study. The alkaloid content of E + plants was significantly higher than that of E- plants. Gao and Nan (2007) believed that endophytic fungi can significantly increase the alkaloid content of plants, which is consistent with this result.

Under different concentrations of alkali stress, endophytic fungi had a significant effect on the flavonoid content in the stems of *H. bogdanii*. The flavonoid content in E + plants was significantly lower than that in E− plants, and the content of flavonoids in high alkali concentrations was significantly higher than that in low alkali concentrations. Chen (2019) suggested that endophytic fungal infection significantly reduced the content of flavonoids in *H.bogdanii*, and the results are similar to those of the present study; salt stress increases the total flavonoid content in roots, stems, leaves, and flowers (Yan. 2011; Hou et al. 2016). Other studies (Zhou et al. 2004) showed that flavonoids accumulate under stress. In this experiment, as the alkali concentration increased, the flavonoid content increased, and the high alkali concentration flavonoid content was significantly higher than the low alkali concentration. This result is similar to that of salt stress, indicating that both salt and alkali stress can increase flavonoid content.

Latch et al. (1985) suggested that the increase in plant biomass of *Lolium perenne L.* infected with endophytic fungi may be related to gibberellin (GA). Wang et al. (2007) reported that gibberellin can inhibit the synthesis of flavonoids by reducing the activity of chalcone synthase (CHS). In the present study, we measured the hormones of E + and E− plants of *H. bogdanii* and found that the content of gibberellin GA3 in E + leaves of *H. bogdanii,* planted indoors and outdoors, was higher than that of E− (data to be published). The higher the content of GA3, the more it inhibited the content of flavonoids, which was consistent with the result that the content of flavonoids in E + plants was significantly lower than that of E-.

Alkaloids are N-based secondary metabolites, and flavonoids are C-based secondary metabolites. According to the hypothesis of "carbon nutrient balance" proposed by Bryant et al. (1983), there is a balance between C-based secondary metabolites (such as terpenes and phenols) and N-based secondary metabolites (such as alkaloids). In the present study, endophytic fungi significantly promoted the content of N-based secondary metabolites alkaloids, but significantly reduced the content of C-based secondary metabolites flavonoids to maintain the nutrient balance in the plant. The experimental results also align with the "carbon nutrient balance" hypothesis.

In summary, endophytic fungi promote the increase of phosphorus content by promoting acid phosphatase activity in plant leaves, and the effect is more significant under alkali stress. Endophytic fungi increased plant polyphenol content and polyphenol oxidase activity under alkali stress, and plant polyphenol oxidase activity decreased gradually with an increase in alkali concentration. Endophytic fungi could significantly increase alkaloid content in plants, but alkali treatment decreased alkaloid content in plants. With an increase of alkali concentration, flavonoid content increased, and flavonoid contents under high alkali concentrations were significantly higher those under low alkali concentrations. However, endophytic fungi decreased the content of plant flavonoids. Endophytic fungi could have significantly promoted the alkaloid contents of secondary metabolites, based on N, and significantly reduced flavone contents, based on carbon, as a strategy of maintaining nutritional balance in plants.

Therefore, the mechanism via which endophytic fungi improve alkali tolerance of host plants influences secondary metabolites. Endophytic fungi can improve the alkali resistance of host plants by increasing the contents of secondary metabolites, polyphenols and alkaloids, and enhancing polyphenol oxidase and acid phosphatase activities, which in turn increase phosphorus contents in plants. The results of the present study could facilitate the enhancement of alkali tolerance in plants.

## Data Availability

The data are real and valid, and the material is easily accessible.
